# Crystal structure of 3-benzyl-1-[(1,2,3,4-tetra­hydro­naphthalen-1-yl­idene)amino]­thio­urea

**DOI:** 10.1107/S2056989015021064

**Published:** 2015-11-21

**Authors:** Shaaban K. Mohamed, Joel T. Mague, Mehmet Akkurt, Alaa A Hassan, Ahmed T. Abdel-Aziz, Mustafa R. Albayati

**Affiliations:** a Faculty of Science & Engineering, School of Healthcare Science, Manchester Metropolitan University, Manchester M1 5GD, England; b Chemistry Department, Faculty of Science, Minia University, 61519 El-Minia, Egypt; c Department of Chemistry, Tulane University, New Orleans, LA 70118, USA; d Department of Physics, Faculty of Sciences, Erciyes University, 38039 Kayseri, Turkey; e Kirkuk University, College of Education, Department of Chemistry, Kirkuk, Iraq

**Keywords:** crystal structure, thio­semicarabazides, anti­proliferative agents

## Abstract

In the title compound, C_18_H_19_N_3_S, the dihedral angle between the planes of the benzene rings is 58.63 (8)°. The six-membered ring bonded to the thio­semicarbazide group (r.m.s. deviation = 0.038 Å) adopts a sofa conformation, with one of the methyl­ene-group C atoms as the flap. A short intra­molecular N—H⋯N contact is observed. In the crystal, mol­ecules are linked by weak N—H⋯S inter­actions to generate *C*(4) chains propagating in the [010] direction, with adjacent mol­ecules related by glide symmetry.

## Related literature   

For the anti­tumour activities of thio­semicarbazides, see: Vandresen *et al.* (2014 ▸); Xie *et al.* (2014 ▸); Gan *et al.* (2014 ▸). For the synthesis of the title compound, see: Mague *et al.* (2014 ▸)
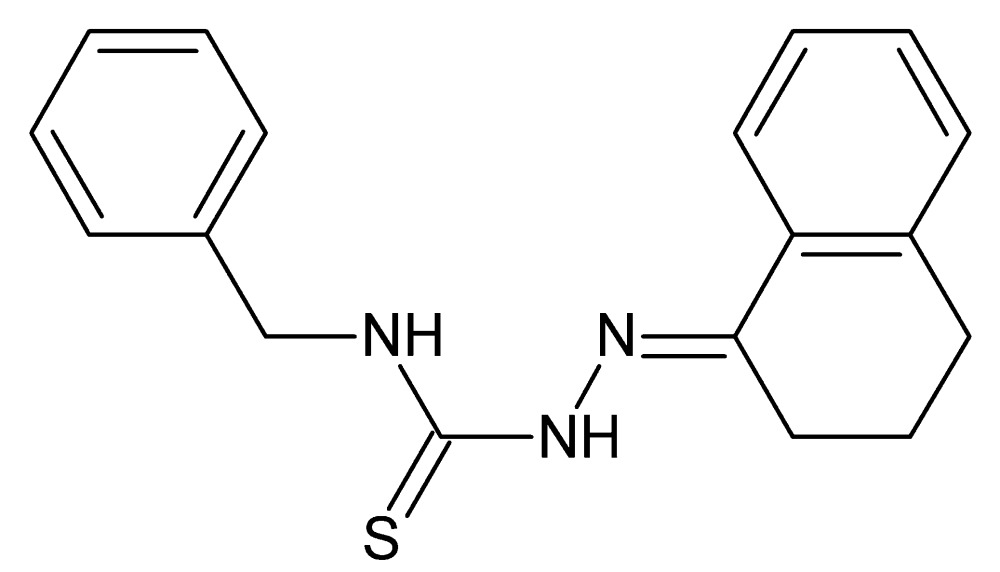


## Experimental   

### Crystal data  

C_18_H_19_N_3_S
*M* *_r_* = 309.42
Orthorhombic, 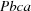

*a* = 11.9129 (5) Å
*b* = 9.6914 (4) Å
*c* = 27.8220 (11) Å
*V* = 3212.1 (2) Å^3^
*Z* = 8
Cu *K*α radiation
μ = 1.77 mm^−1^
*T* = 150 K
0.22 × 0.18 × 0.05 mm

### Data collection  

Bruker D8 VENTURE PHOTON 100 CMOS diffractometer
Absorption correction: multi-scan (*SADABS*; Bruker, 2014 ▸) *T*_min_ = 0.84, *T*_max_ = 0.91
61936 measured reflections
3155 independent reflections
2795 reflections with *I* > 2σ(*I*)
*R*_int_ = 0.050

### Refinement  

*R*[*F* ^2^ > 2σ(*F* ^2^)] = 0.035
*wR*(*F* ^2^) = 0.098
*S* = 1.06
3155 reflections
199 parameters
H-atom parameters constrained
Δρ_max_ = 0.56 e Å^−3^
Δρ_min_ = −0.22 e Å^−3^

### 

Data collection: *APEX2* (Bruker, 2014 ▸); cell refinement: *SAINT* (Bruker, 2014 ▸); data reduction: *SAINT*; program(s) used to solve structure: *SHELXT* (Bruker, 2014 ▸); program(s) used to refine structure: *SHELXL2014* (Sheldrick, 2015 ▸); molecular graphics: *DIAMOND* (Brandenburg & Putz, 2012 ▸); software used to prepare material for publication: *SHELXTL* (Bruker, 2014 ▸).

## Supplementary Material

Crystal structure: contains datablock(s) global, I. DOI: 10.1107/S2056989015021064/hb7536sup1.cif

Structure factors: contains datablock(s) I. DOI: 10.1107/S2056989015021064/hb7536Isup2.hkl

Supporting information file. DOI: 10.1107/S2056989015021064/hb7536Isup3.cml

**Fig. 1.**
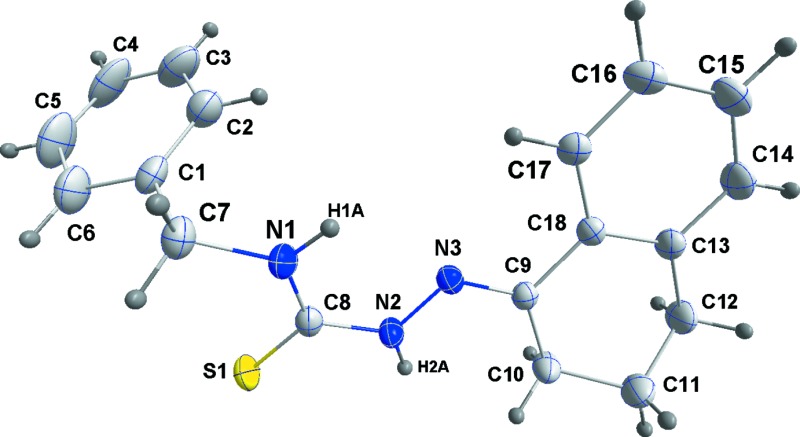
The title molecule with 50% probability displacement ellipsoids.

**Fig. 2.**
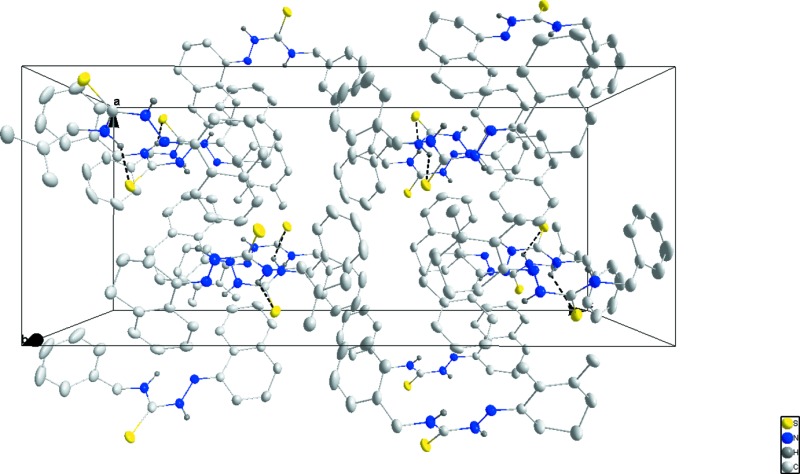
Packing viewed down the b axis. N—H···S interactions are shown by dotted lines.

. DOI: 10.1107/S2056989015021064/hb7536fig1.tif
The title mol­ecule with 50% probability displacement ellipsoids.

b . DOI: 10.1107/S2056989015021064/hb7536fig2.tif
Packing viewed down the *b* axis. N—H⋯S inter­actions are shown by dotted lines.

CCDC reference: 1435397

Additional supporting information:  crystallographic information; 3D view; checkCIF report

## Figures and Tables

**Table 1:** Hydrogen-bond geometry (Å, °)

*D*—H⋯*A*	*D*—H	H⋯*A*	*D*⋯*A*	*D*—H⋯*A*
N1—H1*A*⋯N3	0.91	2.20	2.6219 (16)	108
N1—H1*A*⋯S1^i^	0.91	2.85	3.5790 (13)	138

Symmetry code: (i) 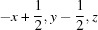.

## supplementary crystallographic information

### S1. Comment

Recently, several kinds of thiosemicarbazone derivatives were synthesized and their antitumor activities were also reported (Vandresen *et al.*, 2014; Xie *et al.*, 2014; Gan *et al.*, 2014). As part of our studies in this area, we report here the synthesis and crystal structural determination of the title compound.

In the title molecule (Fig. 1), the dihedral angle between the phenyl ring (C1–C6) and the aromatic portion of the tetrahydronaphthylidene unit (C13–C18) is 58.64 (5)°. A Cremer-Pople analysis of the conformation of the C9–C13,C18 ring gave puckering parameters Q = 0.434 (2) Å, θ = 126.2 (2)° and φ = 305.1 (2)°. The molecules pack in chains running parallel to the *b* axis assisted by weak N1—H1A···S1^i^ (i: 1/2 - *x*, -1/2 + *y*, *z*) interactions (Table 1, Fig. 2).

### S2. Experimental

The title compound was prepared according to our recently reported method (Mague *et al.*, 2014). The product was recrystallized from ethanol solution to afford colorless tablets (90% yield) *M*.p. 413 - 414 K.

### S3. Refinement

H atoms attached to C atoms were placed in calculated positions (C—H = 0.95–0.99 Å) while those attached to N atoms were placed in locations derived from a difference map and their parameters adjusted to give N—H = 0.91 Å. All were included as riding contributions with isotropic displacement parameters 1.2–1.5 times those of the attached atoms.

### Crystal data

**Table:**

C_18_H_19_N_3_S	*D*_x_ = 1.280 Mg m^−^^3^
*M**_r_* = 309.42	Cu *K*α radiation, λ = 1.54178 Å
Orthorhombic, *P**b**c**a*	Cell parameters from 9005 reflections
*a* = 11.9129 (5) Å	θ = 4.9–72.0°
*b* = 9.6914 (4) Å	µ = 1.77 mm^−^^1^
*c* = 27.8220 (11) Å	*T* = 150 K
*V* = 3212.1 (2) Å^3^	Tablet, colourless
*Z* = 8	0.22 × 0.18 × 0.05 mm
*F*(000) = 1312	

### Data collection

**Table:**

Bruker D8 VENTURE PHOTON 100 CMOS diffractometer	3155 independent reflections
Radiation source: INCOATEC IµS micro–focus source	2795 reflections with *I* > 2σ(*I*)
Mirror monochromator	*R*_int_ = 0.050
Detector resolution: 10.4167 pixels mm^-1^	θ_max_ = 72.2°, θ_min_ = 3.2°
ω scans	*h* = −14→14
Absorption correction: multi-scan (*SADABS*; Bruker, 2014)	*k* = −11→11
*T*_min_ = 0.84, *T*_max_ = 0.91	*l* = −34→34
61936 measured reflections	

### Refinement

**Table:**

Refinement on *F*^2^	Primary atom site location: structure-invariant direct methods
Least-squares matrix: full	Secondary atom site location: difference Fourier map
*R*[*F*^2^ > 2σ(*F*^2^)] = 0.035	Hydrogen site location: mixed
*wR*(*F*^2^) = 0.098	H-atom parameters constrained
*S* = 1.06	*w* = 1/[σ^2^(*F*_o_^2^) + (0.0522*P*)^2^ + 1.4441*P*] where *P* = (*F*_o_^2^ + 2*F*_c_^2^)/3
3155 reflections	(Δ/σ)_max_ = 0.001
199 parameters	Δρ_max_ = 0.56 e Å^−^^3^
0 restraints	Δρ_min_ = −0.22 e Å^−^^3^

### Special details

**Table:**

Geometry. All e.s.d.'s (except the e.s.d. in the dihedral angle between two l.s. planes) are estimated using the full covariance matrix. The cell e.s.d.'s are taken into account individually in the estimation of e.s.d.'s in distances, angles and torsion angles; correlations between e.s.d.'s in cell parameters are only used when they are defined by crystal symmetry. An approximate (isotropic) treatment of cell e.s.d.'s is used for estimating e.s.d.'s involving l.s. planes.
Refinement. Refinement of *F*^2^ against ALL reflections. The weighted *R*-factor *wR* and goodness of fit *S* are based on *F*^2^, conventional *R*-factors *R* are based on *F*, with *F* set to zero for negative *F*^2^. The threshold expression of *F*^2^ > σ(*F*^2^) is used only for calculating *R*-factors(gt) *etc*. and is not relevant to the choice of reflections for refinement. *R*-factors based on *F*^2^ are statistically about twice as large as those based on *F*, and *R*- factors based on ALL data will be even larger. H-atoms attached to carbon were placed in calculated positions (C—H = 0.95 - 0.99 Å) while those attached to nitrogen were placed in locations derived from a difference map and their parameters adjusted to give N—H = 0.91 Å. All were included as riding contributions with isotropic displacement parameters 1.2 - 1.5 times those of the attached atoms.

### Fractional atomic coordinates and isotropic or equivalent isotropic displacement parameters (Å^2^)

**Table:**

	*x*	*y*	*z*	*U*_iso_*/*U*_eq_	
S1	0.08381 (3)	0.69807 (4)	0.37402 (2)	0.02936 (13)	
N1	0.23130 (10)	0.48914 (12)	0.37501 (4)	0.0250 (3)	
H1A	0.2789	0.4340	0.3581	0.030*	
N2	0.18664 (10)	0.57805 (12)	0.30147 (4)	0.0243 (3)	
H2A	0.1458	0.6361	0.2827	0.029*	
N3	0.27037 (9)	0.49802 (12)	0.28232 (4)	0.0223 (3)	
C1	0.29867 (14)	0.56296 (15)	0.45561 (5)	0.0287 (3)	
C2	0.41047 (15)	0.57894 (19)	0.44281 (6)	0.0388 (4)	
H2	0.4386	0.5343	0.4149	0.047*	
C3	0.48124 (18)	0.6596 (2)	0.47050 (7)	0.0524 (5)	
H3	0.5578	0.6695	0.4616	0.063*	
C4	0.4413 (2)	0.7256 (2)	0.51086 (7)	0.0588 (6)	
H4	0.4900	0.7817	0.5296	0.071*	
C5	0.3306 (2)	0.7102 (2)	0.52401 (7)	0.0578 (6)	
H5	0.3030	0.7551	0.5520	0.069*	
C6	0.25919 (18)	0.62890 (19)	0.49641 (5)	0.0421 (4)	
H6	0.1829	0.6185	0.5056	0.051*	
C7	0.22186 (13)	0.47002 (15)	0.42688 (5)	0.0286 (3)	
H7A	0.2394	0.3727	0.4347	0.034*	
H7B	0.1433	0.4878	0.4367	0.034*	
C8	0.17257 (12)	0.58125 (14)	0.35015 (5)	0.0231 (3)	
C9	0.27768 (11)	0.49111 (13)	0.23608 (5)	0.0206 (3)	
C10	0.19577 (13)	0.55643 (16)	0.20165 (5)	0.0298 (3)	
H10A	0.2161	0.6546	0.1971	0.036*	
H10B	0.1196	0.5531	0.2158	0.036*	
C11	0.19402 (14)	0.48477 (18)	0.15289 (6)	0.0356 (4)	
H11A	0.1620	0.3911	0.1564	0.043*	
H11B	0.1459	0.5374	0.1304	0.043*	
C12	0.31195 (14)	0.47477 (18)	0.13267 (5)	0.0339 (4)	
H12A	0.3099	0.4231	0.1020	0.041*	
H12B	0.3404	0.5687	0.1258	0.041*	
C13	0.39089 (12)	0.40340 (15)	0.16706 (5)	0.0247 (3)	
C14	0.48145 (13)	0.32664 (16)	0.14988 (6)	0.0305 (3)	
H14	0.4926	0.3188	0.1162	0.037*	
C15	0.55529 (13)	0.26181 (16)	0.18071 (6)	0.0328 (3)	
H15	0.6154	0.2082	0.1683	0.039*	
C16	0.54132 (12)	0.27535 (15)	0.22997 (6)	0.0299 (3)	
H16	0.5928	0.2325	0.2514	0.036*	
C17	0.45229 (12)	0.35136 (14)	0.24798 (5)	0.0242 (3)	
H17	0.4436	0.3613	0.2817	0.029*	
C18	0.37478 (11)	0.41390 (13)	0.21678 (5)	0.0206 (3)	

### Atomic displacement parameters (Å^2^)

**Table:**

	*U*^11^	*U*^22^	*U*^33^	*U*^12^	*U*^13^	*U*^23^
S1	0.0343 (2)	0.0285 (2)	0.0252 (2)	0.00308 (14)	0.00802 (14)	−0.00293 (13)
N1	0.0291 (6)	0.0258 (6)	0.0200 (6)	−0.0001 (5)	0.0013 (5)	−0.0034 (5)
N2	0.0271 (6)	0.0254 (6)	0.0204 (6)	0.0045 (5)	0.0021 (5)	−0.0016 (5)
N3	0.0219 (6)	0.0218 (6)	0.0232 (6)	0.0000 (4)	0.0021 (4)	−0.0022 (4)
C1	0.0406 (8)	0.0263 (7)	0.0192 (6)	−0.0016 (6)	−0.0040 (6)	0.0049 (6)
C2	0.0412 (9)	0.0445 (9)	0.0308 (8)	−0.0055 (8)	−0.0046 (7)	0.0048 (7)
C3	0.0518 (11)	0.0630 (12)	0.0425 (10)	−0.0206 (10)	−0.0188 (9)	0.0151 (9)
C4	0.0905 (16)	0.0536 (12)	0.0325 (10)	−0.0305 (12)	−0.0287 (10)	0.0096 (8)
C5	0.0977 (18)	0.0514 (12)	0.0244 (8)	−0.0166 (12)	−0.0052 (10)	−0.0066 (8)
C6	0.0599 (11)	0.0423 (9)	0.0242 (7)	−0.0045 (8)	0.0025 (8)	−0.0027 (7)
C7	0.0368 (8)	0.0273 (7)	0.0217 (7)	−0.0036 (6)	0.0024 (6)	0.0023 (6)
C8	0.0248 (7)	0.0235 (7)	0.0209 (6)	−0.0058 (5)	0.0023 (5)	−0.0023 (5)
C9	0.0220 (7)	0.0183 (6)	0.0214 (6)	−0.0013 (5)	0.0002 (5)	−0.0013 (5)
C10	0.0322 (8)	0.0321 (8)	0.0250 (7)	0.0105 (6)	−0.0022 (6)	−0.0015 (6)
C11	0.0356 (8)	0.0438 (9)	0.0275 (8)	0.0066 (7)	−0.0063 (6)	−0.0026 (7)
C12	0.0364 (9)	0.0453 (9)	0.0202 (7)	0.0052 (7)	0.0011 (6)	−0.0011 (6)
C13	0.0248 (7)	0.0248 (7)	0.0244 (7)	−0.0029 (6)	0.0024 (5)	−0.0022 (5)
C14	0.0297 (8)	0.0322 (8)	0.0297 (7)	−0.0028 (6)	0.0098 (6)	−0.0042 (6)
C15	0.0243 (7)	0.0279 (7)	0.0463 (9)	0.0019 (6)	0.0096 (7)	−0.0046 (7)
C16	0.0232 (7)	0.0248 (7)	0.0417 (8)	0.0014 (6)	−0.0010 (6)	0.0028 (6)
C17	0.0234 (7)	0.0220 (7)	0.0270 (7)	−0.0026 (6)	−0.0002 (5)	0.0007 (6)
C18	0.0208 (6)	0.0171 (6)	0.0239 (7)	−0.0028 (5)	0.0017 (5)	−0.0007 (5)

### Geometric parameters (Å, º)

**Table:**

S1—C8	1.6855 (14)	C9—C18	1.4786 (18)
N1—C8	1.3285 (19)	C9—C10	1.5069 (19)
N1—C7	1.4592 (17)	C10—C11	1.524 (2)
N1—H1A	0.9098	C10—H10A	0.9900
N2—C8	1.3651 (18)	C10—H10B	0.9900
N2—N3	1.3713 (16)	C11—C12	1.516 (2)
N2—H2A	0.9099	C11—H11A	0.9900
N3—C9	1.2913 (18)	C11—H11B	0.9900
C1—C6	1.385 (2)	C12—C13	1.509 (2)
C1—C2	1.387 (2)	C12—H12A	0.9900
C1—C7	1.512 (2)	C12—H12B	0.9900
C2—C3	1.384 (3)	C13—C14	1.395 (2)
C2—H2	0.9500	C13—C18	1.4004 (19)
C3—C4	1.377 (3)	C14—C15	1.380 (2)
C3—H3	0.9500	C14—H14	0.9500
C4—C5	1.377 (3)	C15—C16	1.387 (2)
C4—H4	0.9500	C15—H15	0.9500
C5—C6	1.391 (3)	C16—C17	1.385 (2)
C5—H5	0.9500	C16—H16	0.9500
C6—H6	0.9500	C17—C18	1.405 (2)
C7—H7A	0.9900	C17—H17	0.9500
C7—H7B	0.9900		
			
C8—N1—C7	124.03 (12)	C9—C10—C11	112.53 (12)
C8—N1—H1A	116.9	C9—C10—H10A	109.1
C7—N1—H1A	119.0	C11—C10—H10A	109.1
C8—N2—N3	119.18 (12)	C9—C10—H10B	109.1
C8—N2—H2A	119.4	C11—C10—H10B	109.1
N3—N2—H2A	121.0	H10A—C10—H10B	107.8
C9—N3—N2	117.74 (12)	C12—C11—C10	110.29 (13)
C6—C1—C2	119.00 (16)	C12—C11—H11A	109.6
C6—C1—C7	120.17 (15)	C10—C11—H11A	109.6
C2—C1—C7	120.77 (14)	C12—C11—H11B	109.6
C3—C2—C1	120.34 (18)	C10—C11—H11B	109.6
C3—C2—H2	119.8	H11A—C11—H11B	108.1
C1—C2—H2	119.8	C13—C12—C11	111.79 (12)
C4—C3—C2	120.4 (2)	C13—C12—H12A	109.3
C4—C3—H3	119.8	C11—C12—H12A	109.3
C2—C3—H3	119.8	C13—C12—H12B	109.3
C5—C4—C3	119.80 (18)	C11—C12—H12B	109.3
C5—C4—H4	120.1	H12A—C12—H12B	107.9
C3—C4—H4	120.1	C14—C13—C18	118.90 (13)
C4—C5—C6	120.06 (19)	C14—C13—C12	120.60 (13)
C4—C5—H5	120.0	C18—C13—C12	120.50 (13)
C6—C5—H5	120.0	C15—C14—C13	121.51 (14)
C1—C6—C5	120.40 (19)	C15—C14—H14	119.2
C1—C6—H6	119.8	C13—C14—H14	119.2
C5—C6—H6	119.8	C14—C15—C16	119.66 (14)
N1—C7—C1	113.61 (12)	C14—C15—H15	120.2
N1—C7—H7A	108.8	C16—C15—H15	120.2
C1—C7—H7A	108.8	C17—C16—C15	119.98 (14)
N1—C7—H7B	108.8	C17—C16—H16	120.0
C1—C7—H7B	108.8	C15—C16—H16	120.0
H7A—C7—H7B	107.7	C16—C17—C18	120.62 (14)
N1—C8—N2	115.89 (12)	C16—C17—H17	119.7
N1—C8—S1	125.21 (10)	C18—C17—H17	119.7
N2—C8—S1	118.90 (11)	C13—C18—C17	119.28 (13)
N3—C9—C18	116.15 (12)	C13—C18—C9	120.18 (12)
N3—C9—C10	124.61 (12)	C17—C18—C9	120.54 (12)
C18—C9—C10	119.23 (12)		
			
C8—N2—N3—C9	176.26 (12)	C9—C10—C11—C12	−52.80 (18)
C6—C1—C2—C3	0.0 (3)	C10—C11—C12—C13	55.55 (19)
C7—C1—C2—C3	−177.27 (16)	C11—C12—C13—C14	149.52 (14)
C1—C2—C3—C4	−0.4 (3)	C11—C12—C13—C18	−30.9 (2)
C2—C3—C4—C5	0.6 (3)	C18—C13—C14—C15	−0.3 (2)
C3—C4—C5—C6	−0.4 (3)	C12—C13—C14—C15	179.30 (15)
C2—C1—C6—C5	0.2 (3)	C13—C14—C15—C16	−1.5 (2)
C7—C1—C6—C5	177.49 (16)	C14—C15—C16—C17	1.3 (2)
C4—C5—C6—C1	0.0 (3)	C15—C16—C17—C18	0.8 (2)
C8—N1—C7—C1	−87.70 (17)	C14—C13—C18—C17	2.3 (2)
C6—C1—C7—N1	136.73 (15)	C12—C13—C18—C17	−177.28 (13)
C2—C1—C7—N1	−46.0 (2)	C14—C13—C18—C9	−178.21 (13)
C7—N1—C8—N2	−175.61 (13)	C12—C13—C18—C9	2.2 (2)
C7—N1—C8—S1	4.1 (2)	C16—C17—C18—C13	−2.6 (2)
N3—N2—C8—N1	−9.87 (18)	C16—C17—C18—C9	177.96 (13)
N3—N2—C8—S1	170.43 (10)	N3—C9—C18—C13	−178.67 (13)
N2—N3—C9—C18	174.84 (11)	C10—C9—C18—C13	0.82 (19)
N2—N3—C9—C10	−4.6 (2)	N3—C9—C18—C17	0.80 (19)
N3—C9—C10—C11	−155.52 (14)	C10—C9—C18—C17	−179.72 (13)
C18—C9—C10—C11	25.04 (19)		

### Hydrogen-bond geometry (Å, º)

**Table:**

*D*—H···*A*	*D*—H	H···*A*	*D*···*A*	*D*—H···*A*
N1—H1*A*···N3	0.91	2.20	2.6219 (16)	108
N1—H1*A*···S1^i^	0.91	2.85	3.5790 (13)	138

Symmetry code: (i) −*x*+1/2, *y*−1/2, *z*.
