# Supplementary figures and images for: Crystal structure of 3-benzyl-1-[(1,2,3,4-tetra­hydro­naphthalen-1-yl­idene)amino]­thio­urea
Source: Acta Crystallogr E Crystallogr Commun. 2015 Nov 21;71(Pt 12):o974–5. doi: 10.1107/S2056989015021064 (PMC4719930; doi:10.1107/S2056989015021064)

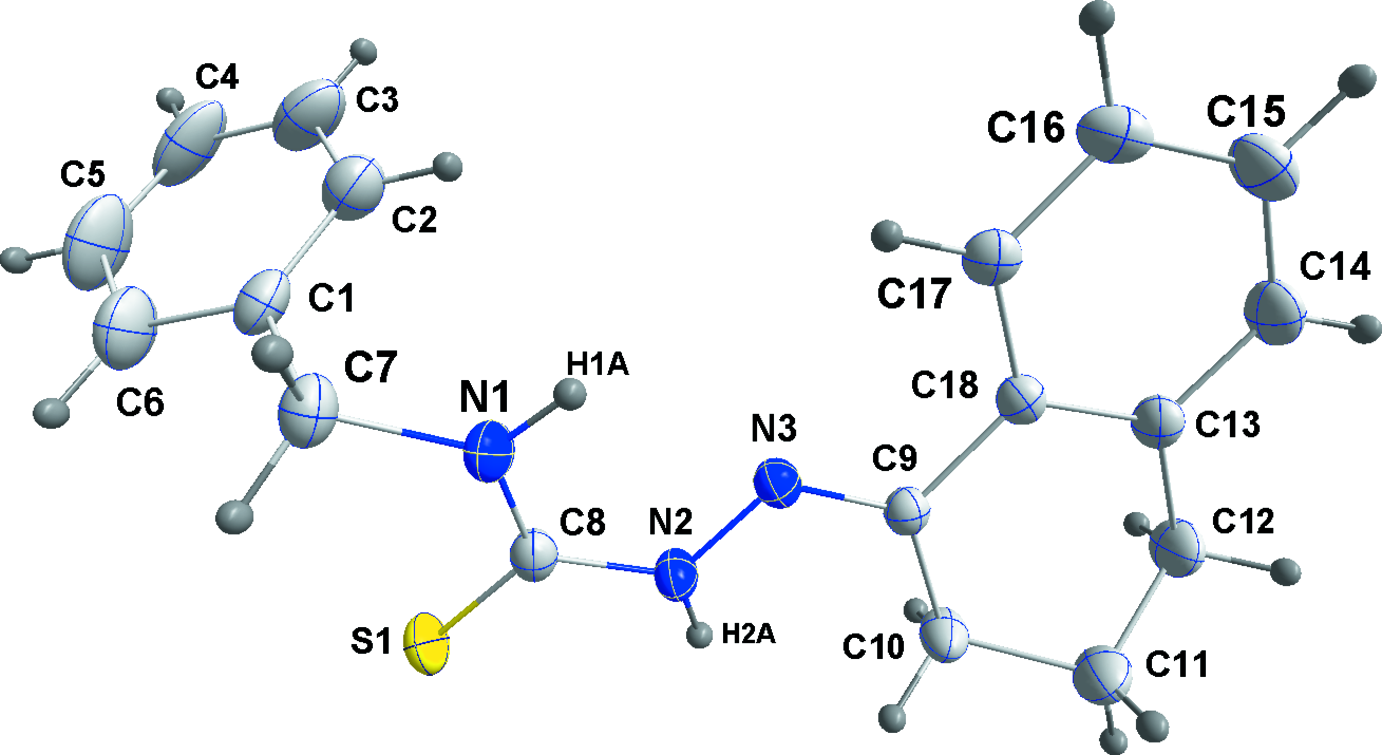

Supplement: Supplementary file 4 [file e-71-0o974-fig1.tif]

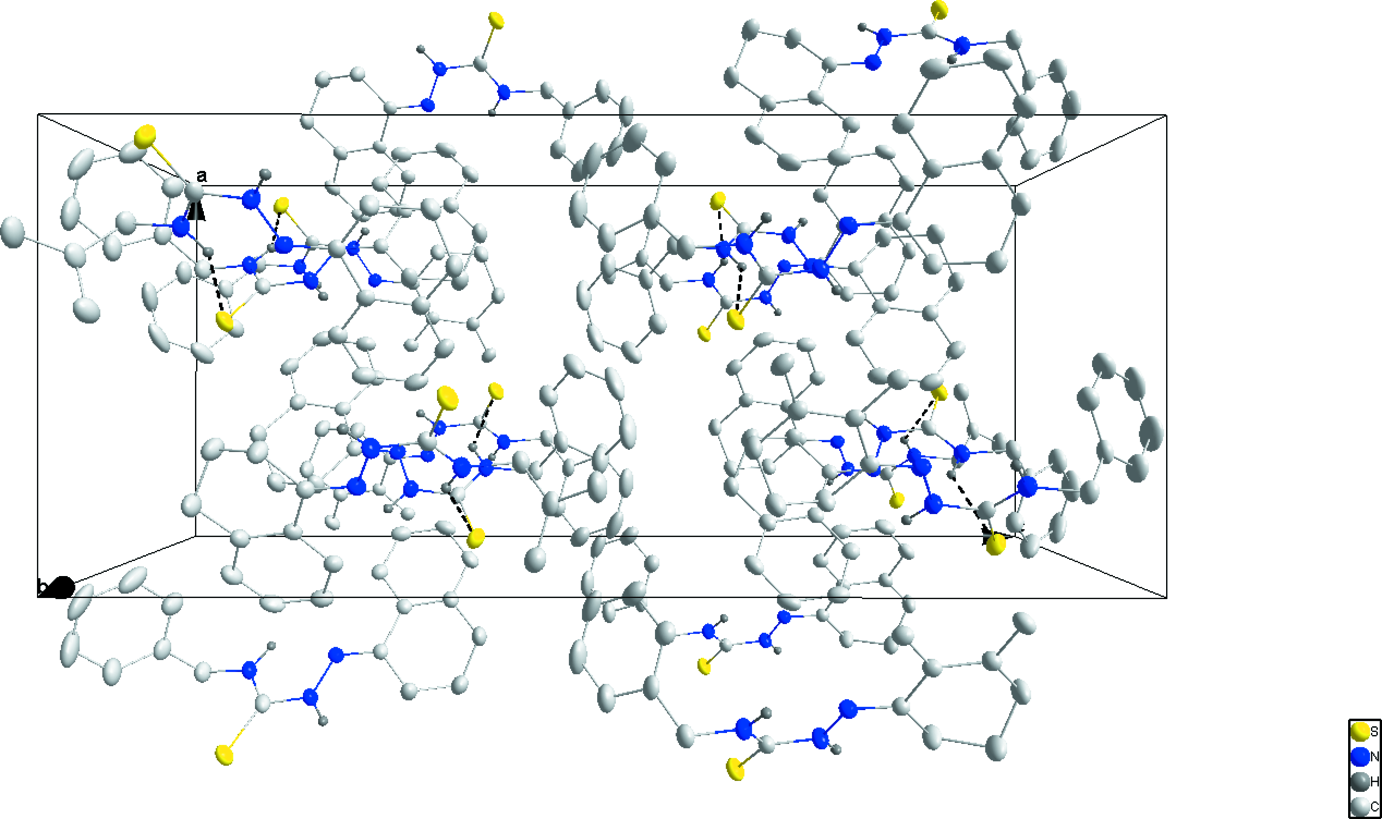

Supplement: Supplementary file 5 [file e-71-0o974-fig2.tif]
